# In-Hospital Disease Burden of Sarcoidosis in Switzerland from 2002 to 2012

**DOI:** 10.1371/journal.pone.0151940

**Published:** 2016-03-17

**Authors:** Susanne Pohle, Florent Baty, Martin Brutsche

**Affiliations:** Department of Pulmonary Medicine, Cantonal Hospital St. Gallen, St. Gallen, Switzerland; Wexner Medical Center at The Ohio State University, UNITED STATES

## Abstract

Sarcoidosis is a multisystem disease with an unpredictable and sometimes fatal course while the underlying pathomechanism is still unclear. Reasons of the increasing hospitalization rate and mortality in the United States remain in dispute but incriminated are a number of distinct comorbidities and risk factors as well as the application of more aggressive therapeutic agents. Studies reflecting the recent development in central Europe are lacking. Our aim was to investigate the recent mortality and hospitalization rates as well as the underlying comorbidities of hospitalized sarcoidosis patients in Switzerland. In this longitudinal, nested case-control study, a nation-wide database provided by the Swiss Federal Office for Statistics enclosing every hospital entry covering the years 2002–2012 (*n* = 15,627,573) was analyzed. There were 8,385 cases with a diagnosis of sarcoidosis representing 0.054% (8,385 / 15,627,573) of all hospitalizations in Switzerland. These cases were compared with age- and sex-matched controls without the diagnosis of sarcoidosis. Hospitalization and mortality rates in Switzerland remained stable over the observed time period. Comorbidity analysis revealed that sarcoidosis patients had significantly higher medication-related comorbidities compared to matched controls, probably due to systemic corticosteroids and immunosuppressive therapy. Sarcoidosis patients were also more frequently re-hospitalized (median annual hospitalization rate 0.28 [IQR 0.15-0.65] vs. 0.19 [IQR 0.13-0.36] per year; *p* < 0.001), had a longer hospital stay (6 [IQR 2-13] vs. 4 [IQR 1-8] days; *p* < 0.001), had more comorbidities (4 [IQR 2-7] vs. 2 [IQR 1-5]; *p* < 0.001), and had a significantly higher in-hospital mortality (2.6% [95% CI 2.3%-2.9%] vs. 1.8% [95% CI 1.5%-2.1%] (*p* < 0.001). A worse outcome was observed among sarcoidosis patients having co-occurrence of associated respiratory diseases. Moreover, age was an important risk factor for re-hospitalization.

## Introduction

Sarcoidosis is a systemic disease of unknown cause resolving in complete remission in many cases but sometimes leading to substantial and even fatal organic dysfunctions [1]. Nowadays, novel and more aggressive therapeutic agents are used to optimize the treatment of sarcoidosis patients, although their benefit is discussed controversially [1]. To date, there are only few studies reflecting the recent development of the mortality and hospitalization rates of sarcoidosis in Europe. In the United States, the number of hospitalizations and the rate of mortality among sarcoidosis patients have substantially increased over the last decades [2, 3]. The cause of this trend is unknown and not associated with an increasing incidence of sarcoidosis. Incriminated are a number of distinct complications namely pulmonary hypertension, comorbidities (e.g. diabetes mellitus, heart failure) as well as important risk factors like race, age and sex [3–7]. The frequent use of systemic corticosteroids and immunosuppressive therapy might also lead to higher infection rates and associated complications. Furthermore, sarcoidosis has been associated with a higher risk for cancer and pulmonary embolism [8, 9].

The aim of this study was to investigate the hospitalization rate and in-hospital mortality among sarcoidosis patients over the last years in Switzerland, as well as the associated comorbidities and risk factors of this disease.

## Materials and Methods

### Hospitalization Database

All our observations were derived from a dataset provided by the Swiss Federal Office for Statistics, which offers a nation-wide coverage of all hospitalized patients since 1998. In this database, the patient information is fully anonymized. No written informed consent was given to the patients who were unidentifiable due to the anonymization. The data belong to the Swiss Federal Office for Statistics (Bundesamt für Statistik, Neuchâtel, Switzerland) who provides regulated access to the data for research purpose. In analogy to the study of Deubelbeiss and colleagues [10] or Baty and collaborators [11], data since 2002 were extracted for the analysis, since satisfactory coding quality was reached by then. These data were imported into a relational SQL database (MySQL, version 5.6.24). The database was interfaced with the R statistical software (version 3.1.2) [12], using the dedicated package RMySQL. It included 15,627,573 entries (6,337,575 unique patients) corresponding to all hospitalized cases in Switzerland between 2002 and 2012. Each patient in this database was identified uniquely so that it was possible to track re-hospitalizations of every patient. The database included geographical and temporal information (patient’s residency, hospitalization by canton, year and month of hospitalization, length of hospital stay) as well as age at admission and reason/type of discharge (including death). The patients’ diagnosis list included one main diagnosis as well as up to 50 additional diagnoses coded using the International Classification of Disease version 10 (ICD-10) codes (http://www.who.int/classifications/icd/en/). The coding version ICD-10 was uniformly used all along the study time period.

### Nested Case-control Study Design

For the current study, sarcoidosis was defined by the ICD-10 code D86*. The cases were defined as any hospitalizations with the diagnosis of sarcoidosis either as main or concomitant diagnosis. Geriatric, obstetric, psychiatric hospitalization and cases of rehabilitation were excluded from the study. The controls consisted of hospitalizations not including sarcoidosis in the main or concomitant diagnoses. Controls were extracted from the hospitalization database using a random procedure and matching the cases 1:1 for age, gender, and month of hospitalization.

### Comorbidities and Mortality

Sarcoidosis-comorbidities were defined as any additional diagnosis coded by the ICD-10 nomenclature. The Charlson’s comorbidity index [13] was used to assign a predicted mortality score to patients.

### Statistical Analysis

Descriptive statistics were reported as median and inter-quartile-range (IQR). The enrichment of sarcoidosis-associated comorbidities was tested using conditional logistic regression in order to account for the way the controls were sampled in our nested case-control design. Results were reported as odds-ratio (OR) with 95% confidence intervals (CI) together with *p*-values from likelihood ratio tests. Time to first re-hospitalization was defined as the time elapsed between the first and second hospitalization record of a given sarcoidosis patient in the database. Data from patients without re-hospitalization, i.e. recorded only once in the database, were censored (lost to follow-up censoring). Time—to—event data were analyzed using Kaplan-Meier estimates, log-rank tests and Cox-proportional hazards regression. Results were reported as hazard ratio (HR) with 95% confidence intervals (CI) together with the Wald test statistic. Hazard ratios provide the percentage of increase in the hazard rates for an increase of one unit in the explanatory variable. Generalized estimating equations (using working independence correlation structure) was used to test associations with one or several covariates while accounting for within-patient variability.

Principal component analysis (PCA) was used to explore correlations among sarcoidosis-associated comorbidities. PCA depicts how comorbidities are related to each other and how sarcoidosis cases are grouped according to comorbidities. A data-matrix summarizing the presence/absence (1/0) of comorbidities in sarcoidosis cases was created. Scaled PCA was applied to the comorbidity matrix. The role played by external explanatory variables, including age, length-of-hospital stay, gender, in-hospital death, hospitalization rate, and number of comorbidities, was further explored using vector fitting procedure to identify the directions of maximal correlation with the external variables in the PCA space [14]. PCA results were directly displayed using biplot representation [15]. PCA biplots are organized as follows: due to the very large number of hospitalization cases, smoothed color density image was used to depict the density of cases in the biplot representation (PCA scores); comorbidities were represented using framed labels (PCA loadings); external variables were superimposed to the biplot using vector representations (arrows) pointing to the direction of most rapid change. The length of the arrows is proportional to the correlation between the PCA configuration and the explanatory variable.

All analyses were done at the hospitalization level, except for the re-hospitalization analysis which was done at the patient level. All statistical computations were done using the R statistical software (version 3.1.2) [12]. The following extension packages were used: survival, geepack, Epi, multipleNCC, ade4 and vegan.

## Results

### Characteristics of Sarcoidosis Hospitalizations in Switzerland

The number of hospitalized cases with a diagnosis of sarcoidosis either as main or secondary diagnosis between 2002 and 2012 was 8,385 corresponding to 0.054% (8385 / 15,627,573) of all hospitalizations in Switzerland, and representing 4,664 unique patients (Table 1). Overall, 50.2% were male, and the median age was 58 years (IQR: 45–70).

**Table 1:** Baseline characteristics of hospitalized cases with sarcoidosis compared to age- and sex-matched controls without diagnosis of sarcoidosis (nested case-control design).

	Sarcoidosis	Control	*p*-value
Number of hospitalizations (n)	8,385	8,385	-
Median age in years [IQR]	58 [45–70]	matched	-
Sex ratio (male/female)	50.2	matched	-
Unique patients (n)	4,664	8,366	-
Median Hospitalization rate /year [IQR]	0.28 [0.15–0.65]	0.19 [0.13–0.36]	< 0.001
Median time between 2 hosp in days [IQR]	167.5 [61–456]	730 [31–1095]	< 0.001
Median length-of-stay in days [IQR]	6 [2–13]	4 [1–8]	< 0.001
Median number of comorbidities [IQR]	4 [2–7]	2 [1–5]	< 0.001
% patients without comorbidities [95% CI]	11 [11–12]	32 [31–33]	< 0.001
% in-hospital death [95% CI]	2.6 [2.3–2.9]	1.8 [1.5–2.1]	< 0.001

The proportion of hospitalizations due to sarcoidosis was age-dependent. A higher percentage of hospitalization was found among patients aged 40–50 years (1%) and elderly patients defined as more than 70 year old (6.3%). Re-hospitalizations occurred 1.80 times per patient on average (8,385 hospitalizations in 4,664 patients).

In 30% of cases, sarcoidosis was the main reason for hospitalization (primary diagnosis).

**Fig 1:**
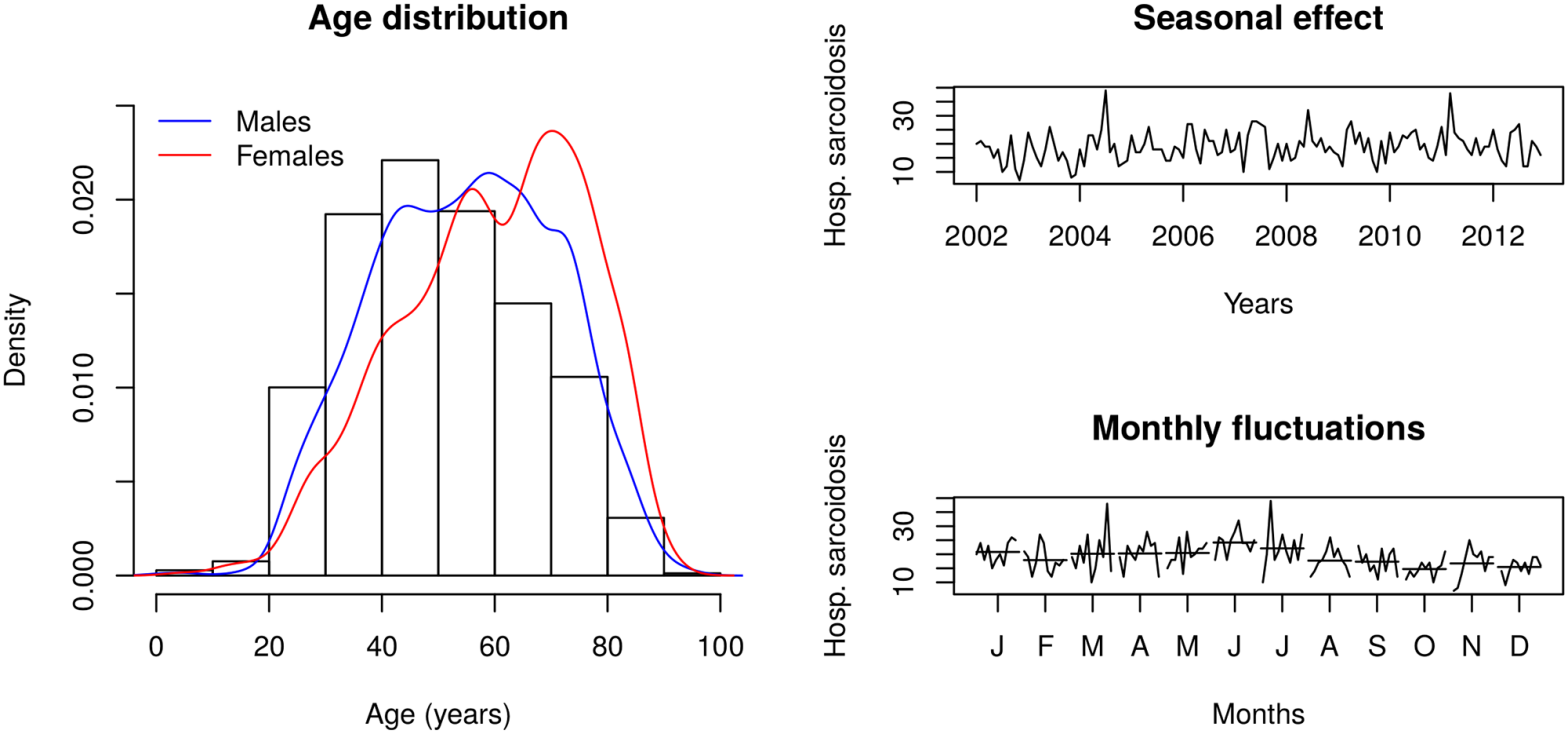
Age, gender and seasonal distribution of hospitalized cases with sarcoidosis.

A seasonal pattern was found over the observational period with the highest rates of hospitalizations in June and the lowest in October and December (*p* < 0.001) (Fig 1).

**Fig 2:**
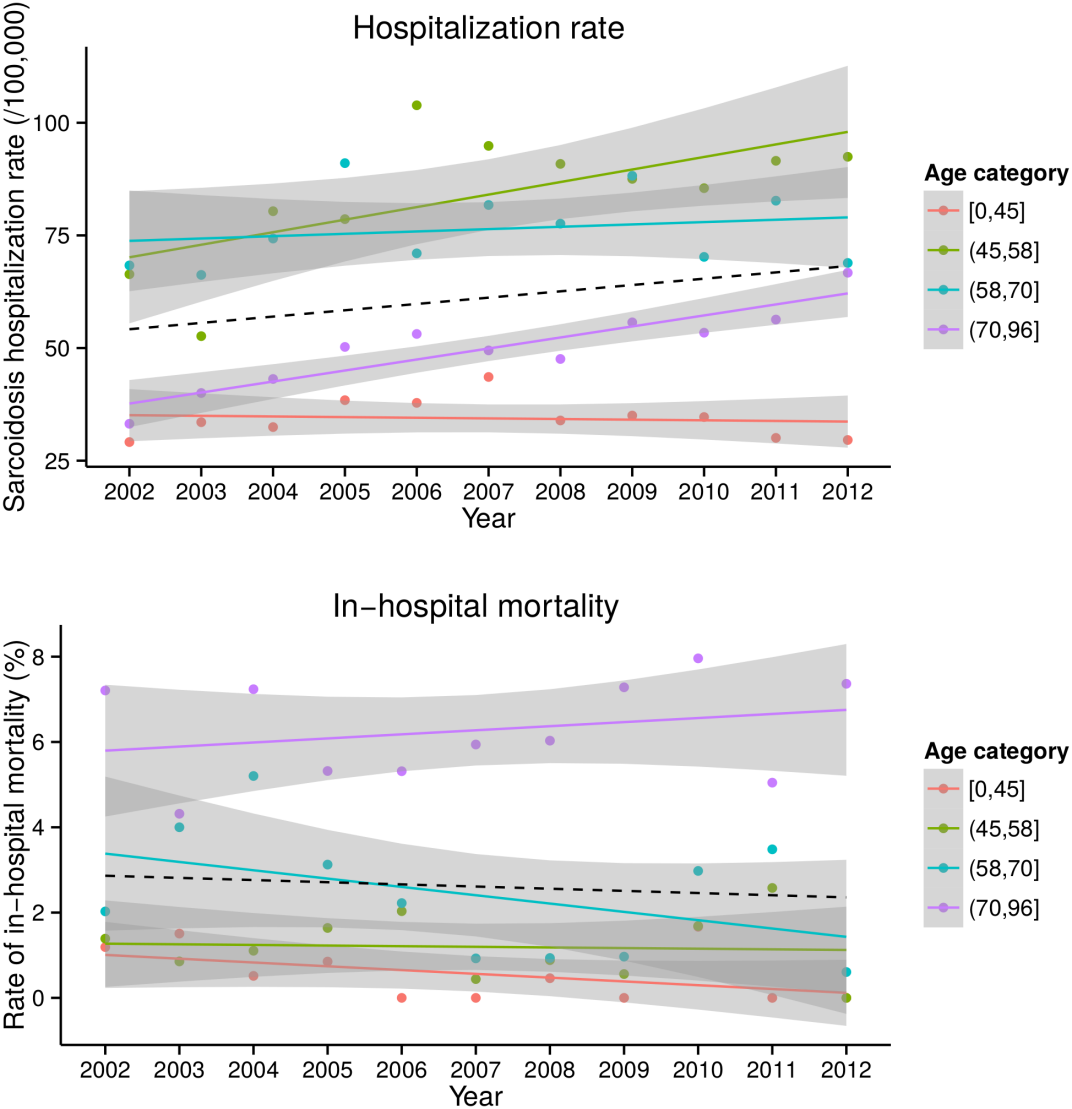
Hospitalization rate and in-hospital mortality among sarcoidosis patients over time. The upper panel shows the hospitalization rate of sarcoidosis patients between 2002 and 2012 (per 100,000 hospitalizations) displayed for 4 age categories. The lower panel depicts the in-hospital mortality of sarcoidosis patients between 2002 and 2012 subdivided into 4 age categories. The black dashed line in the upper and lower panels represents the overall evolution of the hospitalization rate and in-hospital mortality rate over time, respectively.

Fig 2 displays the rate of hospitalization in sarcoidosis patients (upper panel) together with the rate of in-hospital mortality (lower panel) over the time period 2002–2012. Overall, both the rate of hospitalization and the in-hospital mortality remained stable. However, there was an increased rate of hospitalization in the age categories (45–58] (*p* < 0.001) and (70–96] (*p* < 0.001). The in-hospital mortality rate (lower panel) did not increase significantly in any age category over the observed time period (2002–2012), although there was a trend towards an increase of cases in the older category (age >70) (*p* = 0.085) (Fig 2).

### Comparison of Sarcoidosis Cases vs. Controls

The characteristics of sarcoidosis cases and matched controls are summarized in Table 1. Overall, sarcoidosis cases had a significantly worse outcome compared to controls: they were more frequently rehospitalized (median annual hospitalization rate 0.28 [IQR 0.15–0.65] vs. 0.19 [IQR 0.13–0.36] per year; *p* < 0.001), had a longer hospital stay (6 [IQR 2–13] vs. 4 [IQR 1–8] days; *p* < 0.001), had more comorbidities (4 [IQR 2–7] vs. 2 [IQR 1–5]; *p* < 0.001), and had a significantly higher in-hospital mortality (2.6% [95% CI 2.3%-2.9%] vs. 1.8% [95% CI 1.5%-2.1%] (*p* < 0.001).

### Comorbidities among Sarcoidosis Cases

Among 4,096 observed comorbidities, 529 were significantly either enriched or under-represented in sarcoidosis cases (*p* < 0.05). Of these, 57 with the lowest *p*-values (*p* < 1 × 10^−10^) were kept in the analysis (Table 2). Besides well-known sarcoidosis-related manifestations and complications including respiratory failure, interstitial pulmonary disease, pulmonary hypertension and kidney diseases (J960, J961, J841, J969, I270, I279, I2728, L52, N162, N189, N19, R590, R591, G532, H221, G473, E559, E835), we also found a significantly higher declaration of medication and possible medication-related comorbidities (I10, K219, E139, J189, J151, B441, E274, M819, M814, D90, Z922, Z512, Y579, Y420). Furthermore, sarcoidosis patients were associated with other pulmonary diagnosis such as asthma and COPD (J459, J450, J47, J449, J448).

**Table 2:** Prevalence (in percentage) of the most significantly over-/under-represented comorbidities in patients with sarcoidosis (Prev. sarcoidosis) compared to age- and sex-matched patients without diagnosis (Prev. control).

ICD-10	Description	Prev. sarcoidosis	Prev. controls	Odds-ratio [95% CI]	*p*-value
I2728	Other secondary pulmonary hypertension	1.50	0.18	8.5 [5.0–14.7]	<0.001
J961	Chronic respiratory failure	1.80	0.17	12.0 [6.9–20.8]	<0.001
K219	Gastro-oesophageal reflux disease without oesophagitis	2.00	0.76	2.7 [2.0–3.5]	<0.001
N189	Chronic kidney disease	3.00	0.98	3.2 [2.5–4.2]	<0.001
G473	Sleep apnoea	2.40	0.52	4.8 [3.5–6.7]	<0.001
Y579	Drug or medicament	2.70	0.69	4.2 [3.1–5.7]	<0.001
L52	Erythema nodosum	1.00	0.01	88.8 [12.4–638.8]	<0.001
I10	Essential (primary) hypertension	15.00	8.70	2.0 [1.8–2.0]	<0.001
E1390	Other specified diabetes mellitus: Without complications	0.76	0.12	6.7 [3.4–13.1]	<0.001
J841	Other interstitial pulmonary diseases with fibrosis	1.10	0.12	9.7 [5.0–18.7]	<0.001
G998	Other specified disorders of nervous system in diseases classified elsewhere	1.20	0.00	Inf [26-Inf]	<0.001
J984	Other disorders of lung	0.72	0.05	16.7 [6.0–46.6]	<0.001
J459	Asthma	2.80	0.78	3.7 [2.8–4.9]	<0.001
R591	Generalized enlarged lymph nodes	0.50	0.01	42.9 [5.9–312.2]	<0.001
G532	Multiple cranial nerve palsies in sarcoidosis	0.66	0.00	Inf [14-Inf]	<0.001
N162	Renal tubulo-interstitial disorders in blood diseases and disorders involving the immune mechanism	0.80	0.00	Inf [18-Inf]	<0.001
M148	Arthropathies in other specified diseases classified elsewhere	1.30	0.01	110.9 [15.5–795.0]	<0.001
J960	Acute respiratory failure	2.20	0.58	4.0 [2.8–5.5]	<0.001
E139	Other specified diabetes mellitus: Without complications	0.85	0.05	18.1 [6.6–49.8]	<0.001
Y420	Glucocorticoids and synthetic analogues	1.10	0.06	18.1 [7.3–44.6]	<0.001
J189	Pneumonia	2.30	0.56	4.1 [3.0–5.7]	<0.001
I278	Other specified pulmonary heart diseases	0.99	0.16	6.5 [3.6–11.6]	<0.001
R060	Dyspnoea	1.50	0.41	3.8 [2.6–5.6]	<0.001
J969	Respiratory failure	1.30	0.27	4.8 [3.0–7.6]	<0.001
R590	Localized enlarged lymph nodes	1.70	0.16	11.3 [6.4–20.0]	<0.001
M8199	Osteoporosis	1.20	0.31	4.2 [2.7–6.5]	<0.001
J151	Pneumonia due to Pseudomonas	0.57	0.01	48.9 [6.7–354.8]	<0.001
J47	Bronchiectasis	1.10	0.11	10.9 [5.5–21.6]	<0.001
J448	Other specified chronic obstructive pulmonary disease	2.00	0.36	5.9 [4.0–8.7]	<0.001
J450	Predominantly allergic asthma	1.10	0.20	5.4 [3.2–9.1]	<0.001
G4731	Sleep apnoea	1.30	0.30	4.8 [3.1–7.5]	<0.001
Z518	Other specified medical care	1.30	0.32	4.2 [2.7–6.4]	<0.001
E274	Other and unspecified adrenocortical insufficiency	0.57	0.04	30.6 [7.2–130.6]	<0.001
X599	Exposure to unspecified factor causing other and unspecified injury	1.20	2.70	0.44 [0.34–0.55]	<0.001
I270	Primary pulmonary hypertension	2.50	0.24	12.1 [7.5–19.5]	<0.001
D90	Procedure—Radiation therapy	0.66	0.06	11.4 [4.5–28.5]	<0.001
Z512	Other chemotherapy	1.80	0.16	14.1 [7.6–26.0]	<0.001
E559	Vitamin D deficiency	1.10	0.21	5.1 [3.1–8.5]	<0.001
J449	Chronic obstructive pulmonary disease	2.30	0.81	2.9 [2.2–3.9]	<0.001
I279	Pulmonary heart disease	1.20	0.11	12.4 [6.2–24.8]	<0.001
K778	Liver disorders in other diseases classified elsewhere	0.66	0.01	55.0 [7.6–397.4]	<0.001
M8149	Drug-induced osteoporosis	0.60	0.02	26.3 [6.4–108.4]	<0.001
E835	Disorders of calcium metabolism	0.94	0.05	20.1 [7.4–55.1]	<0.001
H221	Iridocyclitis in other diseases classified elsewhere	1.00	0.00	Inf [24-Inf]	<0.001
N19	Unspecified kidney failure	2.00	0.67	3.0 [2.2–4.1]	<0.001
Z470	Follow-up care involving removal of fracture plate and other internal fixation device	0.16	1.30	0.12 [0.07–0.21]	<0.001
Z922	Personal history of long-term (current) use of other medicaments	1.60	0.41	4.0 [2.7–5.8]	<0.001
H259	Senile cataract	0.16	1.10	0.14 [0.08–0.25]	<0.001
S832	Tear of meniscus	0.05	0.81	0.057 [0.02–0.16]	<0.001
M819	Osteoporosis	1.40	0.41	3.6 [2.5–5.3]	<0.001
R942	Abnormal results of pulmonary function studies	0.61	0.05	13.6 [4.9–37.9]	<0.001
B441	Other pulmonary aspergillosis	0.60	0.01	56.5 [7.8–411.6]	<0.001
Z370	Single live birth	0.27	2.10	0.10 [0.06–0.17]	<0.001
O096	Supervision of young primigravida and multigravida	0.04	0.79	0.03 [0.01–0.12]	<0.001
M814	Drug-induced osteoporosis	0.75	0.05	16.3 [5.9–44.8]	<0.001
M233	Other meniscus derangements	0.14	0.82	0.17 [0.09–0.32]	<0.001
F99	Mental disorder	0.02	0.62	0.04 [0.01–0.16]	<0.001

Sarcoidosis cases had a higher rate of pulmonary embolism (1.4% vs. 0.63%; *p* < 0.001) and a lower rate of cancer disease (12% vs. 13%; *p* = 0.002). Under-represented comorbidities among sarcoidosis cases consisted of orthopedical, gestational, geriatric, and mental disorders.

### Sarcoidosis and Pulmonary Embolism

Overall, there were 119 hospitalized cases with sarcoidosis and pulmonary embolism (ICD-10 code: I26*), corresponding to 1.4% of all sarcoidosis hospitalizations. Compared to the control group, sarcoidosis cases had a higher rate of pulmonary embolism (1.4% vs. 0.63%, *p* < 0.001). Cases with the combined diagnosis were older (median age: 71 [IQR 58.5–89.0], *p* < 0.001) and tended to be more frequent among females (sex ratio: 0.42). They had a worse outcome with more comorbidities (median number of comorbidity: 5 [IQR 3–7], *p* < 0.001), a longer in-hospital stay (median length-of-stay: 12 days [IQR 7–18.5], *p* < 0.001) and a higher chance of in-hospital mortality (9.2% [95% CI 4.9–16.3], *p* < 0.001) in comparison to sarcoidosis cases without pulmonary embolism.

### Prognostic Relevance and Correlations of Sarcoidosis-associated Comorbidities

**Fig 3:**
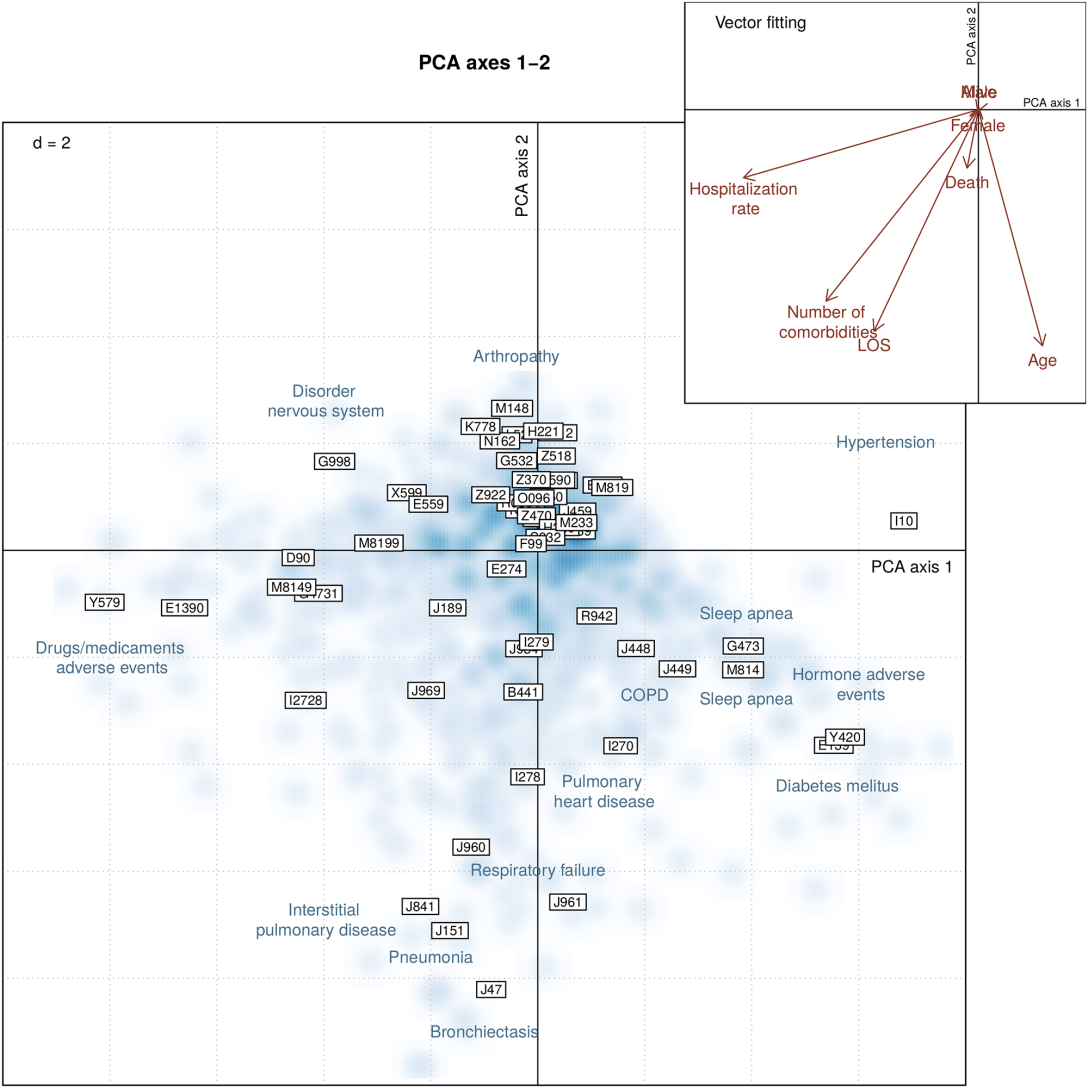
Principal component analysis (PCA) biplot of comorbidities associated with sarcoidosis. Hospitalization cases (PCA scores) are represented using smoothed blue colored density, comorbidities are illustrated by framed labels (PCA loadings). Comorbidities lying in the same direction are correlating with each other, the further away from the center of the plot, the stronger is the influence of comorbidities. The number displayed on the upper left corner indicates the size of the grid. The upper right box represents external variables including age, gender, length-of-stay [LOS], in-hospital mortality [Death], hospitalization rate, and number of comorbidities fitted to the PCA and displayed using vector representation (arrows). The longer the arrow, the stronger the association to the corresponding comorbidities.

Fig 3 displays a PCA biplot showing correlations of comorbidities and the presence of comorbidity clusters among sarcoidosis patients. The comorbidities displayed along the first PCA axis include adverse events related to the use of hormones and medication, as well as diseases including hypertension and diabetes mellitus. Along the second PCA axis, several lung diseases including pneumonia, bronchiectasis, interstitial pulmonary disease, respiratory heart disease, COPD and sleep apnea are lying in the same direction (lower quadrants), thus correlating with each other. In the upper right box, a set of explanatory variables are presented, mostly pointing to the lower direction. These explanatory variables reflecting poorer conditions (higher hospitalization rate, higher number of comorbidities, longer length-of-stay, and older ages) are associated with the presence of concomitant lung diseases.

### Risk Factors for Re-hospitalization

**Fig 4:**
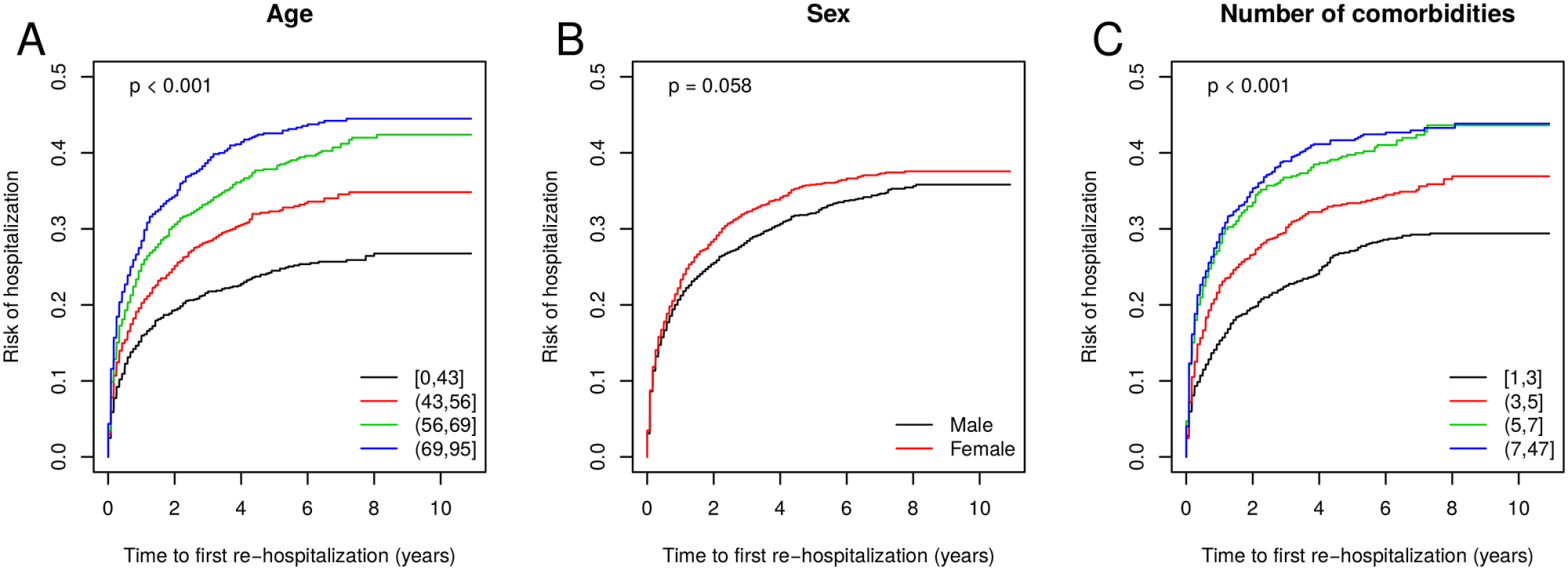
Kaplan-Meier curves of the variables age (A), sex (B) and number of comorbidities (C) influencing the time to first re-hospitalization. For this representation, the explanatory variables age and number of comorbidities were categorized using the quartiles of their distribution. Log-rank test *p*-values are reported in the upper left corner of each panel.

**Fig 5:**
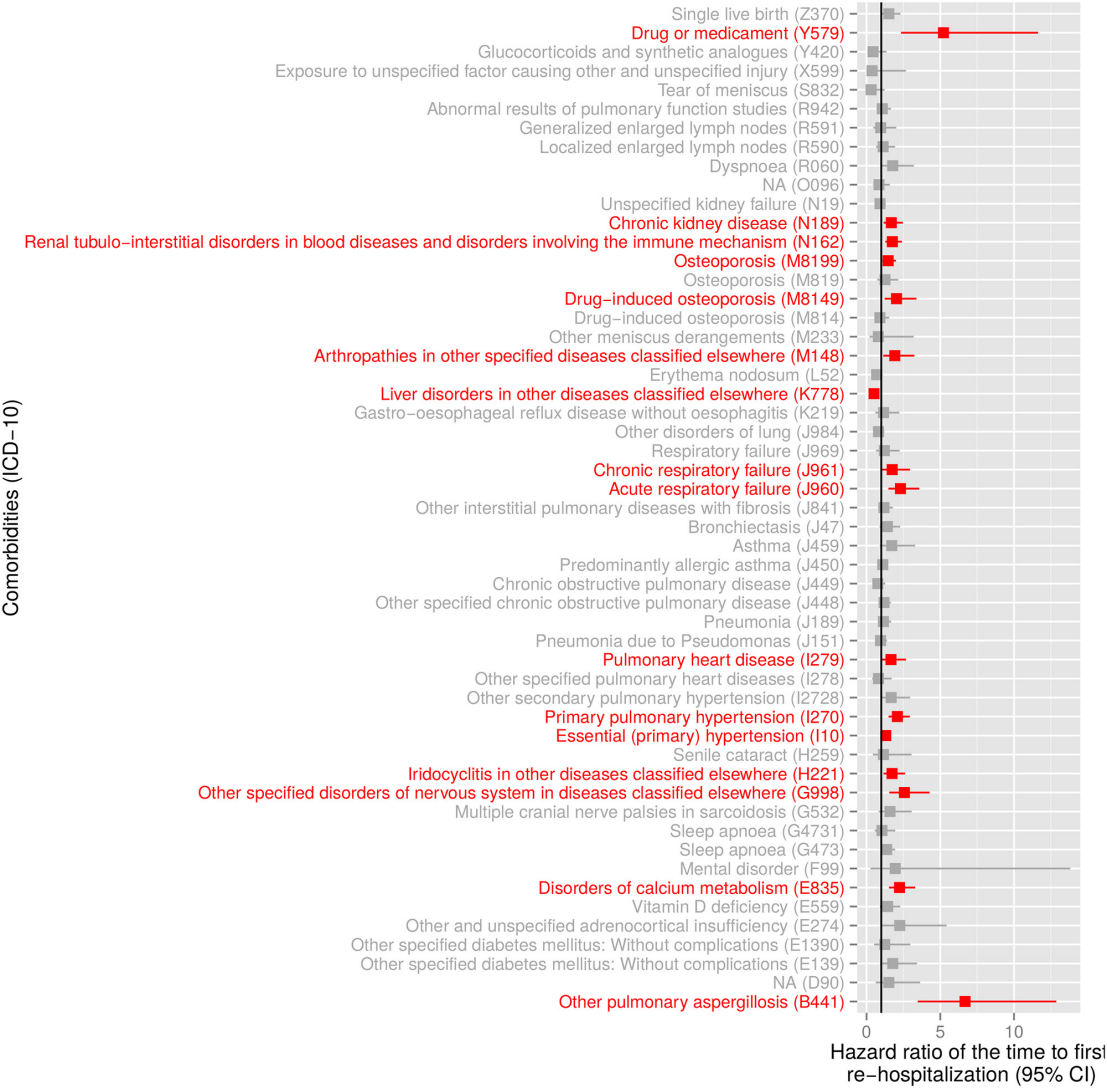
Comorbidities risk factor analysis. The hazard ratios (and 95% confidence intervals) of the time to first re-hospitalization associated with each comorbidity are displayed. Comorbidities that were significantly associated with the time to first re-hospitalization (*p* < 0.05) are depicted in red.

Fig 4 shows the Kaplan-Meier curves representing the association between age, sex and number of comorbidities (panels A, B, C, respectively) and the time to first re-hospitalization. The risk of re-hospitalization was significantly higher in the elderly population with an increase of 1.5% in the hazard rates for each increase of 1 year of age (HR = 1.015 [95% CI: 1.012–1.019], *p* < 0.001). The risk of re-hospitalization was also significantly higher in patients having a larger amount of comorbidities with an increase of 5.4% in the hazard rates for each additional comorbidity (HR = 1.054 [95% CI: 1.042–1.067], *p* < 0.001). No statistically significant difference was found between females and males (HR = 1.104 [95% CI: 0.996–1.223], *p* = 0.058). Fig 5 displays the comorbidities significantly associated with the time to first re-hospitalization. Comorbidities significantly increasing the risk of re-hospitalization in sarcoidosis patients included kidney diseases (N189, N162), osteoporosis (M819, M814), respiratory failure (J960, J961), pulmonary heart disease (I279), pulmonary hypertension (I270) and essential hypertension (I10).

## Discussion

In this longitudinal, nested case-control study, we investigated the recent mortality and hospitalization rates as well as the underlying comorbidities of hospitalized sarcoidosis patients in Switzerland from 2002 to 2012. Furthermore, we analyzed possible risk factors and predictors of outcome compared to matched controls.

Overall, our analysis showed stable mortality and hospitalization rates among sarcoidosis patients in Switzerland over the observed time period. This is in contrast to recent findings of a significant mortality increase, at least for Caucasians, in the United States [4–6]. Reasons could be the comparatively short time period in our study regarding epidemiological changes as well as national differences. On the other hand, the results of the U.K. National Health Service showed concordant results to our study with stable mortality rates over the time period 1979–2008 [16].

By clearly displaying the burden of the disease, our data reveal that sarcoidosis patients showed a significantly worse outcome compared to matched controls regarding re-hospitalization, in-hospital mortality and length of hospital stay. Similar results were recently found when comparing sarcoidosis patients with pedestrian traffic accident patients [6]. In the Black Women’s Health Study, a follow-up study of 59,000 African American women between 1995 and 2009, Tukey and colleagues described a significantly higher mortality rate in women with sarcoidosis in comparison to women without the disease [17]. About 25% of the deaths were directly attributable to the disease itself.

In our study, we identified possible predictors and risk factors associated with a worse outcome. We found that age was a significant prognostic factor of the disease, which is supported by other previous studies [2]. There was an increased in-hospital mortality in sarcoidosis patients older than 70 years and a significant increase regarding the hospitalization rate in the middle and older age categories. Moreover elderly patients had a higher risk of re-hospitalization.

Specific comorbidities possibly contributing to a worse outcome were also detected. For instance, associated respiratory diseases were significantly correlated to a longer hospital stay and a higher number of comorbidities. This defines a phenotype of sarcoidosis patients with a higher morbidity. Among these associated respiratory diseases, there was a higher risk of pulmonary embolism. A link between sarcoidosis and pulmonary embolism was postulated in previous studies [8] while the underlying patho-mechanisms are unclear. We could identify possible predictors of co-occurrence, since hospitalized cases with the combined diagnosis were older, had more comorbidities and were more frequently females. Some previous studies postulated a higher risk for cancer diseases in sarcoidosis patients [18]. Our data from Switzerland showed a slight decrease of cancer diseases in sarcoidosis patients, which is in agreement with the data from a Danish and UK study [19, 20].

Medication-related events seem to substantially affect sarcoidosis patients leading to a significantly higher hospitalization rate as well as to a shorter time to re-hospitalization. Long-term benefits of the corticosteroidal and immunosuppressive therapy are often discussed controversially [1]. Our findings support that these therapeutic agents are sometimes harmful. More attention should be payed to possible therapeutic adverse events and the focus should be put on a close monitoring together with a more restrictive way of administrating medication.

One of the strength of our study is the comprehensive number of patients registered by the Swiss coding system, providing good data quality. In addition, this study is one of a few European studies investigating hospitalizations and mortality rates among sarcoidosis patients and the only one displaying risk factors for re-hospitalization. As a limitation, our analysis is based on a database which is essentially hospitalization-oriented. Our cohort only included hospitalized sarcoidosis cases while a high fraction of patients is typically treated in an out-patient setting. Further information such as medication or laboratory data were not available nor was it possible to contact patients to complete or confirm data.

Overall, the current study is showing stable mortality and hospitalization rates among sarcoidosis patients over the observed time period. However, the burden of the disease becomes apparent, as patients with sarcoidosis show a significantly worse outcome compared to matched controls. This could be due to the disease itself as well as to a cluster of specific comorbidities and complications namely associated respiratory diseases. On the other hand, there is also evidence that side effects of more aggressive treatment approaches could explain this poorer outcome, probably especially affecting patients at older age.

A better knowledge of these prognostic factors could lead to a better understanding of the disease and guide to a more specific monitoring as well as more individualized therapeutic approaches. Further research should be done to evaluate this and maybe contribute to a better outcome.
